# Assessment of Gait Kinematics in the Sagittal Plane in Children with Cerebral Palsy Following Therapy with the PRODROBOT Gait Trainer

**DOI:** 10.3390/s26185708

**Published:** 2026-09-09

**Authors:** Katarzyna Fedejko-Kaflowska, Wiesław Chwała, Krzysztof Kasicki, Łukasz Rydzik, Tadeusz Ambroży, Sławomir Porada

**Affiliations:** 1 Department of Applied Occupational, Therapy Institute of Applied Sciences, University of Physical Culture in Kraków, 31-571 Kraków, Poland; 2 Department of Biomechanics, University of Physical Culture in Kraków, 31-571 Kraków, Poland; wieslaw.chwala@awf.krakow.pl; 3 Department of Sport Theory and Motor Skills, Institute of Sports Sciences, University of Physical Culture in Kraków, 31-571 Kraków, Poland; lukasz.rydzik@awf.krakow.pl (Ł.R.); tadeusz.ambrozy@awf.krakow.pl (T.A.); 4 Faculty of Health Sciences and Psychology, Collegium Medicum, University of Rzeszów, 35-959 Rzeszow, Poland; sporada@ur.edu.pl

**Keywords:** rehabilitation, biomechanics, CP, gait analysis, child development

## Abstract

Background: Gait disturbances are among the most disabling manifestations of cerebral palsy (CP) and substantially affect functional mobility and quality of life. Robot-assisted gait training (RAGT) has been introduced to improve gait performance; however, its effects on lower-limb kinematics remain inconsistent. The aim of this study was to evaluate changes in sagittal-plane gait kinematics in children with CP following a four-week gait rehabilitation program using the PRODROBOT robotic gait trainer. Methods: A prospective quasi-experimental pre–post study with a reference group was conducted. Ten children with spastic cerebral palsy at GMFCS level III aged 8–13 years completed a four-week intervention comprising 20 sessions of robot-assisted gait training. Three-dimensional gait analysis (Vicon) was performed before (KF1) and within 72 h after the end of the intervention (KF2); for each participant, 15 gait cycles were averaged. Eighteen typically developing children aged 7–14 years constituted the normative reference group (CG) and were assessed once. Sagittal plane angular waveforms for the hip, knee, and ankle joints were compared using the difference factor (*f*_1_) and the similarity factor (*f*_2_). Results: The intervention produced selective changes in lower-limb kinematics. At the ankle joint, the range of motion deficit in the sagittal plane relative to the CG decreased from 4.5° to 2.9°, and the difference modulus at 55–60% of the gait cycle decreased from 7.5% to 4.0%. At the knee joint, the difference factor relative to the CG decreased from *f*_1_ = 22.2% to *f*_1_ = 17.4%, with a reduction of the difference modulus by 2–3 percentage points during the loading response phase, terminal stance phase, and terminal swing phase. At the hip joint, the profiles before and after the intervention were respectively *f*_1_ = 2.0% and *f*_2_ = 95.6, and the deviation from the reference pattern remained at the level of *f*_1_ = 54.1% vs. 53.0%; *f*_2_ = 50.2 in both comparisons, with persistent flexion of approximately 10° and a lack of extension in the terminal stance phase. Conclusions: Robot-assisted gait training with the PRODROBOT system produced selective improvements in sagittal-plane gait kinematics in children with cerebral palsy, with the most pronounced effects observed at the ankle joint and, to a lesser extent, the knee. The intervention did not restore a normal gait pattern, particularly at the hip joint. These findings suggest that robotic gait training may serve as a valuable component of comprehensive rehabilitation but should be combined with interventions targeting muscle strength, joint mobility and postural control. Larger randomized studies with long-term follow-up are needed to confirm the clinical effectiveness of this approach.

## 1. Introduction

Cerebral palsy (CP) is a cause of permanent motor disability in childhood. According to the international definition, CP is defined as a group of permanent disorders of the development of movement and posture, causing activity limitation, that are attributed to non-progressive disturbances that occurred in the developing fetal or infant brain; the motor disorders are often accompanied by disturbances of sensation, perception, cognition, communication, and behavior by epilepsy and by secondary musculoskeletal problems [1,2]. The prevalence of CP remains significant despite advances in perinatal care. A recent analysis of registry data showed that in high-income countries, the overall birth prevalence is 1.6 per 1000 live births (95% CI 1.5–1.7), with 1.5 per 1000 (95% CI 1.4–1.6) for CP of pre-/perinatal origin, whereas in low- and middle-income countries, it reaches 3.4 per 1000 (95% CI 3.0–3.9) [3]. Spastic forms of CP account for the majority of cases, and functional classification according to the Gross Motor Function Classification System (GMFCS) remains the primary tool for describing the level of gross motor function [4,5]. Gait disturbances are among the consequences of CP affecting independence and quality of life. They result from primary neurological deficits, including spasticity, muscle weakness, impaired selective motor control and balance, and from secondary musculoskeletal changes that increase with growth [6,7]. Disease-altered gait in CP is most commonly described in the sagittal plane. The role of the sagittal plane in gait analysis stems from the biomechanics of locomotion, specifically through the stance phase, which drives movement. Experimental studies have shown that ankle plantar flexors are the main muscles responsible for body support and the initiation of the swing phase, as well as the muscles capable of regulating the angular momentum of the whole body throughout the stance phase [8,9]. In the pathologically altered gait pattern, these forces are transferred proximally to the hip and knee joints, which may reduce gait economy. For this reason, angular changes of the lower-limb joints in the sagittal plane constitute a clinically important indicator of the effectiveness and efficiency of the gait cycle. The gold standard for gait assessment is three-dimensional gait analysis, which provides kinematic and kinetic data. Reviews and clinical practice guidelines indicate that three-dimensional gait analysis identifies abnormal gait patterns, influences therapeutic decisions, and serves to evaluate the effectiveness of interventions in children with CP [10]. To simplify data interpretation, indices facilitating clinical analysis have been developed, such as the Gait Deviation Index (GDI), which correlates with the GMFCS level [11]. Gait re-education utilizes the principles of motor learning and neuroplasticity: the repetitiveness of movements and task specificity promote motor cortex reorganization and functional improvement [12,13]. Early and goal-directed therapy utilizes the window of greatest neural plasticity in development [14]. This may justify the development of automated gait re-education systems, which allow for a greater number of movement pattern repetitions than passive therapy. Evidence for the effectiveness of robotic-assisted gait training (RAGT), however, remains inconclusive. A meta-analysis by Wang et al. showed that RAGT, compared to conventional rehabilitation, improves gross motor function, balance, and gait endurance, but does not affect gait speed or spasticity [15]. On the other hand, some randomized controlled trials did not confirm the superiority of RAGT over conventional physiotherapy in terms of kinematics and spatiotemporal parameters. A randomized controlled trial by Drużbicki et al., involving 52 children with spastic diplegia, found no improvement in spatiotemporal or kinematic gait parameters after training in the “Lokomat” exoskeleton, where differences between groups in gait speed (*p* = 0.5905) and range of motion (*p* = 0.8676) were non-significant [16]. Body-weight-supported treadmill training also proved to be no more effective than overground walking [17,18]. Promising results were only yielded by next-generation exoskeletons; for instance, in a study by Lerner et al. involving seven children with CP and crouch gait, six out of the seven participants achieved improved knee extension during gait with the exoskeleton after six visits [19]. Current literature emphasizes the need for studies evaluating the impact of robotic devices on kinematic variables in relation to physiological gait [20,21]. This study aimed to evaluate the angular changes in lower-limb joints following PRODROBOT therapy in relation to physiological gait in the sagittal plane in children with CP, filling the gap in evidence regarding the effects of exoskeleton application in the therapeutic process and the analysis of gait changes. This work constitutes the second part of a research project conducted on the same cohort of children with CP. In the first part [22], kinematic profiles in the frontal and transverse planes were analyzed; in this paper, the analysis is limited to the sagittal plane. Both analyses concern disjoint sets of kinematic variables and do not duplicate results. A working hypothesis was formulated that a four-week gait re-education program using the PRODROBOT trainer brings the sagittal angular profiles of the lower-limb joints closer to the reference pattern of healthy peers.

## 2. Materials and Methods

The study was conducted in accordance with the Declaration of Helsinki and approved by the Ethics Committee at The Regional Medical Council in Krakow (Nr 76/KBL/OIL/2024).

### 2.1. Participants Characteristics

#### 2.1.1. Experimental Group

The experimental cohort comprised ten subjects ranging in age from 8 to 13 years (mean age: 9.6 ± 2.45 years). All participants in the experimental group were confirmed to have a diagnosis of cerebral palsy at level III according to the GMFCS scale; there were no other neurological etiologies in the group, making it diagnostically homogeneous. The homogeneous functional level and type of gait pattern limit the influence of clinical diversity on the compared kinematic profiles. Based on the recorded angular profiles in the sagittal plane, a retrospective subclassification of the gait pattern according to Rodda and Graham was performed [23]. A bilateral spastic gait pattern was observed in all participants. The gait pattern was dominated by increased hip and knee flexion during the stance phase with preserved or increased ankle dorsiflexion. The classification was assigned based on the averaged angular curves of each participant in the baseline measurement (KF1) independently by two assessors. Due to the homogeneity of the group in terms of GMFCS level and pattern type, a stratified analysis by gait subtype was not performed. Anthropometric assessments, which utilized motion capture markers to map body segment positions, determined an average height of 1.31 ± 0.13 m. The average body mass was 31.25 ± 9.47 kg, with values ranging from 19.0 to 48.5 kg. Additionally, the mean maximum pelvic width was 0.236 ± 0.03 m, and the baseline body mass index (BMI) averaged 17.63 ± 1.998 kg/m^2^. Comprehensive inclusion criteria are detailed in Table 1. Prior to study commencement, formal informed consent was secured from the legal guardians of all participants, alongside explicit approval from their attending physicians. Inclusion criteria included: (1) age between 8 and 13 years; (2) diagnosis of gait disorders confirmed by medical documentation; (3) absence of contraindications for locomotor training issued by the attending physician; (4) no surgical procedure within the musculoskeletal system within the last 6 months; and (5) a stable pharmacotherapy regimen for a period of at least 4 weeks prior to inclusion in the study. Exclusion criteria consisted of: (1) uncontrolled seizures; (2) severe cognitive deficits preventing cooperation during therapeutic sessions; (3) unstable cardiological or pulmonological conditions; (4) active inflammation or skin wounds within the lower limbs; and (5) concurrent participation in another rehabilitation program aimed at gait improvement. Detailed clinical data of individual participants are presented in Table 1. Before commencing the study, written informed consent was obtained from the legal guardians of all participants, as well as approval from the attending physicians.

#### 2.1.2. Control Group

A normative control group was also established, consisting of 18 healthy volunteers aged 7 to 14 years, with a mean age of 10.1 ± 2.42 years. The baseline anthropometrics for this cohort included an average height of 1.34 ± 0.12 m and a mean body mass of 32.36 ± 7.27 kg (range: 22.4–50.2 kg). Furthermore, the parameter defining maximum pelvic width averaged 0.241 ± 0.028 m, with a mean BMI of 17.85 ± 2.10 kg/m^2^. Inclusion criteria for the control group included: (1) the absence of any musculoskeletal disorders confirmed by clinical examination; (2) no history of lower-limb injuries; (3) no surgical procedures within the last 6 months; (4) no pain complaints regarding the spine or lower limbs; and (5) no intense physical exertion within the 24 h preceding the examination.

The calculated spatiotemporal gait parameters for both the experimental and control cohorts are summarized in Table 1.

### 2.2. Study Design

A prospective, quasi-experimental pre–post study design with a control group was applied. This design involved a two-fold kinematic assessment of the experimental group: immediately before the start of the intervention (KF1) and within 72 h after the completion of the last therapeutic session (KF2). The control group (CG), consisting of peers with typical psychomotor development, underwent a single kinematic assessment to establish reference norms for the physiological gait pattern. The interval between the KF1 and KF2 measurements was four weeks and was short in relation to the maturation rate of the locomotion pattern. In typically developing children, the gait pattern reaches mature characteristics around the age of 7 [24], and all participants were older than this threshold. The increase in body height corresponding to the average growth velocity in this age range is approximately 0.4 cm over four weeks and remains below the uncertainty associated with marker placement; regardless of this, the Plug-in Gait model was calibrated in each session based on the current anthropometric measurements of each participant.

Such a design enabled the evaluation of within-group changes, as well as a comparison of the kinematic profiles of the experimental group with the normative pattern. A 4-week gait re-education program was applied using the fully automated PRODROBOT gait trainer (PRODROMUS, Poland). The dependent variables were the three-dimensional angular waveforms of the lower-limb joints, including the hip, knee, and ankle joints, in the sagittal plane, recorded over the full gait cycle. The analysis was limited to the sagittal plane. The primary endpoints were defined as the change from KF1 to KF2 in the measures of fit of the angular waveform to the CG reference pattern, expressed using the difference factor (F1) and the similarity factor (F2). The secondary endpoints were the change in the range of motion and the qualitative assessment of the difference module profiles in the individual phases of the gait cycle. The experimental and control groups were comparable in terms of anthropometric values (Table 2). Welch’s *t*-tests showed no significant between-group differences regarding age, body height and mass, pelvic width, or BMI (*p* > 0.55; d < 0.25). This allowed us to assume that the observed differences in angular waveforms were not an artifact of differences in body build.

### 2.3. Data Collection Process and Measurement Protocol

Gait kinematics registration was conducted under standardized laboratory conditions, with controlled ambient temperature (22 ± 1 °C) and lighting. Each participant underwent a pre-established preparatory procedure, including familiarization with the laboratory conditions, instruction, and adaptive trials. An optical marker tracking system, Vicon 250 (Vicon Motion Systems Ltd., Oxford, UK), was used for the three-dimensional motion analysis. The system was calibrated according to the manufacturer’s recommendations before each measurement session. The Plug-in Gait biomechanical model (Vicon Nexus) was applied. This model is commonly used in clinical gait analysis and enables the determination of joint angles in three anatomical planes using the Euler/Cardan angle convention. Markers with a diameter of 14 mm were placed on the participants’ skin in accordance with the standard Plug-in Gait model. Before the dynamic registration was started, a static calibration trial was performed in a standing position. Spatiotemporal parameters were calculated automatically in the Vicon Nexus software (v. 2.18) based on gait cycle data, specifically, initial contact with the ground and toe-off detected from marker trajectories. Gait speed (WS) was defined as the mean forward velocity of the pelvis; stride length (STRL) as the distance between consecutive initial contacts of the same limb; stride time (STRT) as the time between them; and cadence (CAD) as the number of steps per unit of time. Double support time (DSAP) and single support time (SSAP), as well as opposite foot off (OFO), were expressed as a percentage of the gait cycle. These parameters were used solely to confirm the spatiotemporal comparability of the groups prior to the analysis of angular waveforms; they were not the subject of kinematic inference.

### 2.4. Description of the Intervention

The intervention consisted of a gait re-education program using the fully automated PRODROBOT robotic trainer by PRODROMUS (Kraków, Poland), designed to control the axial segments of the body during locomotion. This system enables the repetitive execution of step cycles with simultaneous real-time monitoring and correction of the gait pattern, making it a tool designed for both therapeutic and research purposes. The range of motion guidance in the axial segments, the degree of body weight support, and the treadmill belt speed were selected individually based on a preliminary functional assessment of each participant, which included an evaluation of joint range of motion, muscle strength, muscle tone, and gait pattern. The level of support was then progressively modified in subsequent sessions according to the patient’s current capabilities, where the overarching goal was to achieve a total of 10,000 step cycles throughout the entire intervention process while maintaining the participant’s safety and the quality of the gait pattern. Body weight support was initially set at the lowest level, ensuring the maintenance of an upright trunk posture and full foot contact with the ground during the stance phase, and was then reduced in subsequent sessions provided that the quality of the step pattern was maintained. The treadmill speed was gradually increased, on the condition of maintaining the perceived exertion on the Borg scale at a moderate level and the absence of an increase in associated movements. Progression was carried out according to a uniform protocol, and the decision to change parameters was made by the therapist conducting the session based on the ongoing assessment of gait quality. The program consisted of 20 therapeutic sessions conducted over a period of 4 weeks (5 sessions per week, from Monday to Friday). Each session consisted of approximately 500 step cycles, corresponding to 8–10 min of effective walking on the trainer’s treadmill, with a target total of approximately 10,000 step cycles throughout the entire intervention process. During each session, the following parameters were continuously monitored: gait speed, stance and swing phase times, ranges of motion in the axial segments, and the subject’s subjective rating of perceived exertion using the Borg scale. The entire intervention cycle was standardized and conducted by the same trained therapeutic team, specifically two physiotherapists certified in operating the PRODROBOT device, which minimized the variability resulting from the operator factor. For the duration of the intervention, the participants continued their existing rehabilitation program but did not participate in any additional program aimed at improving gait. All sessions were held at a similar time of day to limit the impact of the circadian rhythm on the results.

### 2.5. Statistical Analysis

The subject of the analysis was the waveforms of angular changes in the lower-limb joints. To quantitatively assess the consistency of movement patterns, a two-stage comparative procedure of kinematic profiles was applied. This procedure enabled the determination of the degree of convergence of the individual angular waveforms recorded in the subjects with the reference pattern established based on the control group data. To determine this convergence, two indices were used: the difference factor (*f*_1_) and the similarity factor (*f*_2_). The calculation methods for these indices are presented in Equations (1) and (2) [25,26]. Both indices were model-independent and did not require the description of variable waveforms using a mathematical function. In the calculations, the following factors were applied: the difference factor *f*_1_ and the similarity factor *f*_2_ [25]. Both factors were model-independent, meaning they did not require the description of variable waveforms using a mathematical function. The value of the similarity factor *f*_2_ was expressed using the following equation (Equation (1)):

(1) $$f_{2}=50log\left\{1+{\left[\frac{\sum_{t=1}^{n}{\left(T_{1}-T_{2}\right)}^{2}}{n}\right]}^{\frac{1}{2}}\right\}\;100\;\left[\%\right]$$

Equation (1) Similarity factor value [25,26], where T1 and T2 represent the standardized instantaneous values of, respectively, the COM position, potential, kinetic, and total energy and the energy recovery index, obtained for any two analyzed gait speeds in consecutive one-percent intervals of relative gait cycle time, and where n is the number of tested points at time t. The value of the applied statistic *f*_1_ (difference factor) expresses the percentage difference between two waveform profiles at individual relative time points of the gait cycle and is a measure of the relative difference between any two analyzed curves. In accordance with the principles of applying this statistical procedure, the boundary conditions specified below were adopted [25]; their justification is presented at the end of this section. If the value of the difference factor *f*_1_ fell within the limits of 0 < *f*_1_ < 15 and simultaneously the similarity factor *f*_2_ was close to 100 (but no less than 50), the curves were considered similar. Therefore, for the curves to be considered similar, alongside a high value of the similarity coefficient, they had to be characterized by a difference index of less than 15%. Adopting such a procedure prevents the overly hasty classification of the analyzed waveforms as similar to each other. On the other hand, the use of both indices is a convenient generalization, allowing for the forgoing of a labor-intensive analysis of profile differences for individual phases of the cycle. The difference factor *f*_1_ was defined as follows (Equation (2)):

(2) $$f_{1}=\frac{\sum_{t=1}^{n}\left|T_{1}-T_{2}\right|}{\left|\sum_{t=1}^{n}T_{1}\right|}\;100\;\left[\%\right],$$

Equation (2) Profile difference factor [25,26].

$R\left(t\right)$ denotes the value of the reference profile (the mean profile of the control group CG) at point t of the normalized gait cycle, and $T\left(t\right)$ is the value of the compared profile (KF1 or KF2) at the same point; both quantities are expressed in degrees. The summation runs over all points of the cycle, t=1…n. Each waveform was subjected to time normalization using linear interpolation to 101 equidistant points corresponding to 0–100% of the gait cycle (n=101), while the angular values were left in degrees, without amplitude normalization. In the denominator of the $F_{1}$ factor, the absolute value was calculated for the difference at each point and for the reference value at each point separately ($\Sigma |R\left(t\right)|$). This notation is important for angular data taking negative values, as it prevents the reduction of the denominator when summing values with opposite signs, which would occur in the formulation $\left|\Sigma R\left(t\right)\right|$. No a priori calculation for the sample size was conducted; the sample was a convenience sample, limited by the recruitment window and the anthropometric limits of the PRODROBOT device. With the final experimental group size of n=10, the study is underpowered for confirmatory inference; therefore, null hypothesis significance tests were not applied to the kinematic waveforms. The analysis is descriptive and exploratory in nature. The $F_{1}$ and $F_{2}$ indices were selected as model-independent measures, not requiring a parametric description of the curve nor correction for multiple comparisons at each point of the cycle, which makes them suitable for a small sample where the distributional and cluster assumptions of statistical parametric mapping (SPM) are difficult to meet and the power would be low. We treat statistical parametric mapping and its non-parametric permutational variant as complementary methods for analyzing full waveforms; we indicate their formal application in an adequately powered, diagnostically stratified cohort as a priority for further research. The results should therefore be treated as preliminary and requiring confirmation.

The adopted profile similarity criteria (F1 < 15% and 50 < F2 ≤ 100) correspond to the thresholds defined in the source methodology [25,26] and were applied without modification because the profile values were analyzed in degrees, without prior amplitude normalization. An F2 value > 50 indicates that the root-mean-square difference between corresponding points of the compared profiles is less than approximately 10°, whereas F1 < 15% limits the total magnitude of deviations to less than 15% of the sum of the reference profile values. The simultaneous application of both criteria was necessary because these indices evaluate different aspects of profile similarity. F2 can take high values in the case of curves with a similar shape but shifted relative to each other by a constant value, whereas F1 is sensitive to such a shift but does not account for its location within the cycle. The joint application of both thresholds allows for considering both the agreement of the profile shape and the magnitude of its deviation relative to the reference profile.

## 3. Results

Among all the compared pairs of variables, all met the similarity criterion of 50% < F2 < 100%. Only two pairs of variables met both criteria simultaneously: F1 < 15% and 50% < F2 < 100%. These included KNEE FLEX/EXT_KF2 vs. KNEE FLEX/EXT_KF1 and HIP FLEX/EXT_KF2 vs. HIP FLEX/EXT_KF1. They describe the values of the F1 and F2 indices for the flexion and extension movements of the knee and hip joints. They can therefore be classified as similar curves (Table 3).

Below, the results of the kinematic analysis of the lower-limb joints in the sagittal plane are presented using pairs of graphs, comparing the pre- (KF1) and post-intervention (KF2) states against the reference pattern of the control group (CG), along with a graph illustrating the modulus of the difference profiles of pairwise comparisons of all analyzed curves.

**Figure 1:**
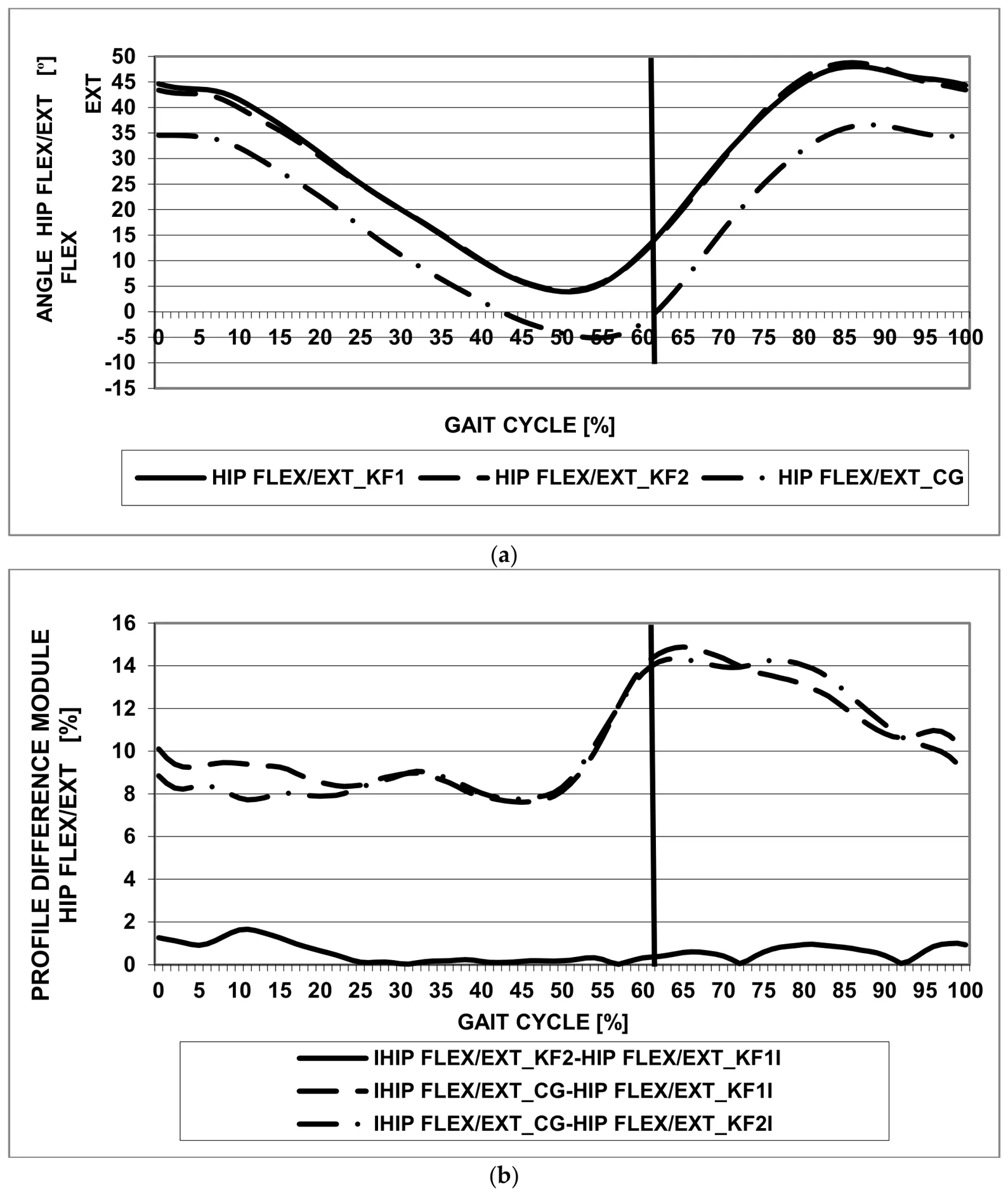
(**a**) Hip flexion/extension curves in the normalized gait cycle and (**b**) difference modulus profiles between hip flexion/extension curves.

As can be seen in Figure 1a, the waveform of the hip flexion/extension angle in the gait cycle for KF1 and KF2 was similar and shifted in all phases towards increased joint flexion relative to the CG group by approx. 10°, and there was an absence of joint hyperextension in the terminal stance (TST) phase. The mean difference between KF2 and CG decreased post-intervention by 1° to 2° in the loading response phase. The hip flexion/extension curve therefore remained in the range of higher values relative to the control group throughout the entire gait cycle, and the post-intervention changes were limited.

**Figure 2:**
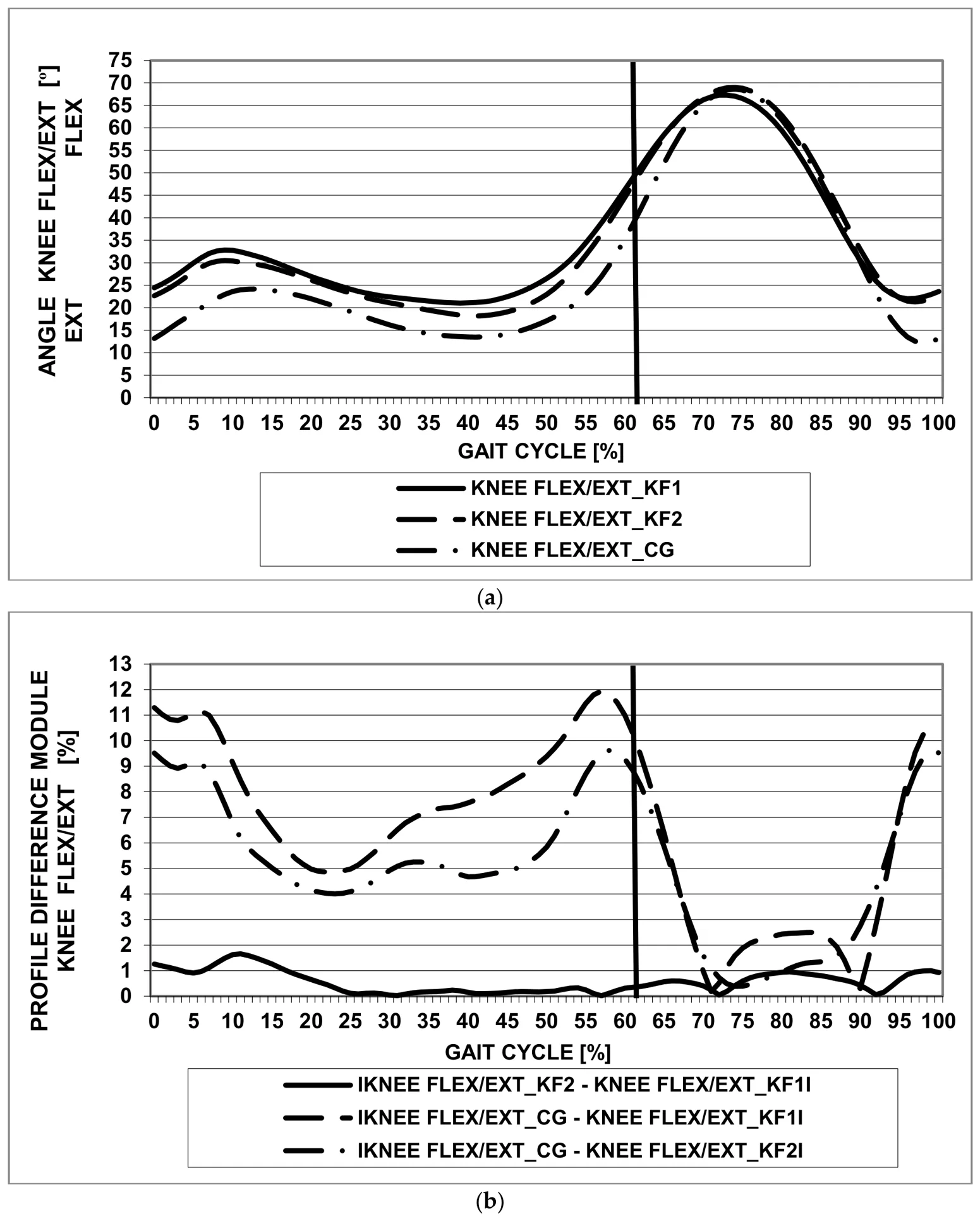
(**a**) Knee flexion/extension curves in the normalized gait cycle and (**b**) difference modulus profiles between knee flexion/extension curves.

Figure 2 presents the flexion/extension angle of the knee joint in the normalized gait cycle.

The KF1 and KF2 curves are characterized by greater knee joint flexion relative to the CG throughout the entire stance phase and in the initial and final swing phases. The largest differences in angle values relative to the CG, reaching 10–12°, were noted in the loading response (LR) phase, the middle part of the terminal stance (TST) phase, and in the terminal swing (TSW). A trend should be pointed out, consisting of a convergence of the range of changes in the KF1 and KF2 curves from 46° to 51° in the stance phase. When the difference moduli are compared (Figure 2b), three areas should be indicated where the largest differences occur between both the KF1 and KF2 curves relative to the CG. These are, respectively, 0–5%, 55–60%, and 95–100% of the cycle, where the highest values of the difference moduli reach the level of 12% for KF1–CG comparisons. Clearly smaller modulus differences of 2–3% were noted for KF2–CG curve comparisons, which corresponded to a decrease in the difference relative to the reference pattern. The KF1 and KF2 curves proved to be the most similar to the changes in the CG group in the mid-swing (MSW) phase. Comparisons of the KF1 and KF2 curves indicate their great similarity, where the value of the difference modulus did not exceed the level of 2%.

**Figure 3:**
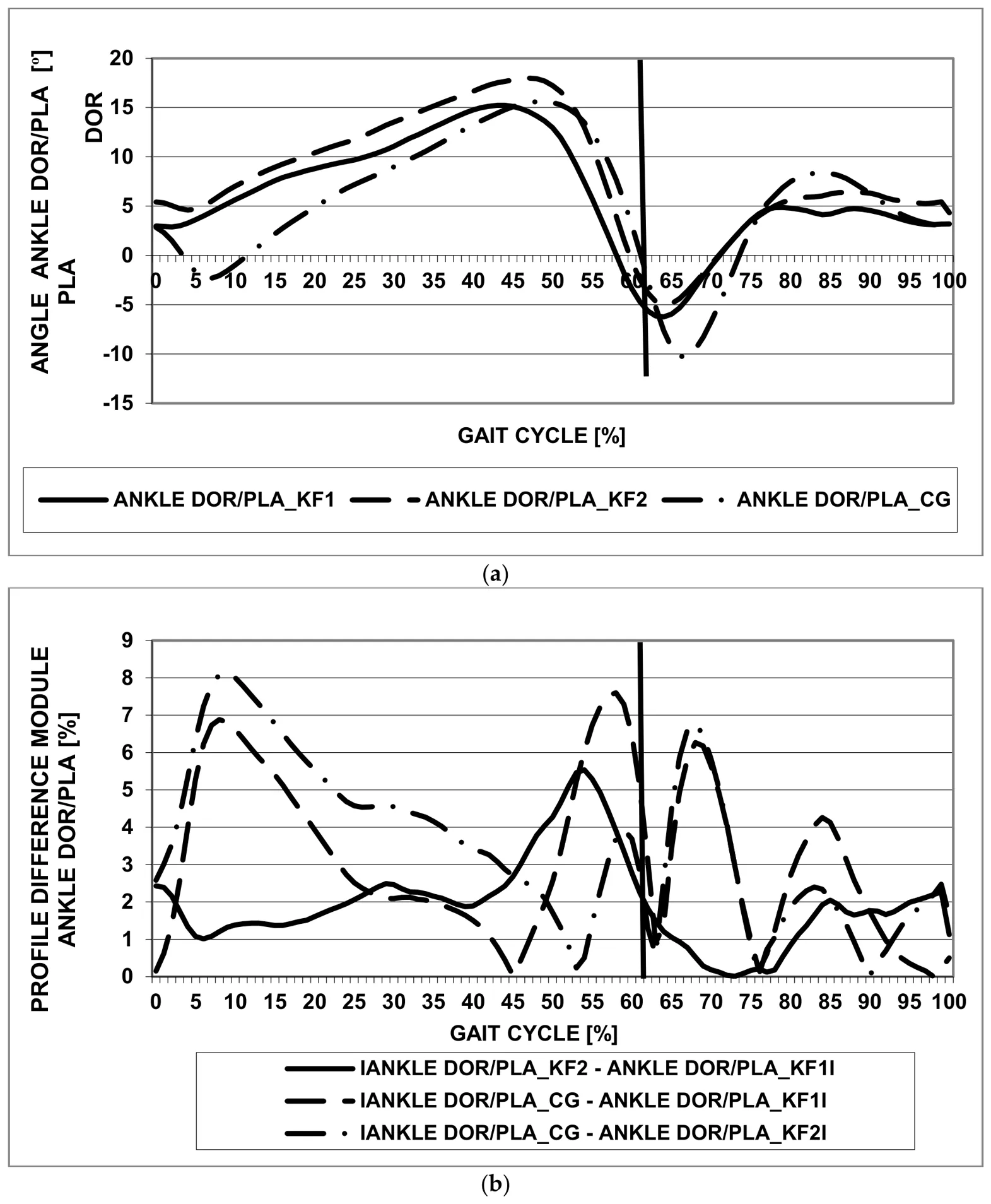
(**a**) Dorsiflexion/plantarflexion curves of the ankle joint in the normalized gait cycle and (**b**) difference modulus profiles between the curves in the ankle joint.

Figure 3a presents the dorsiflexion and plantarflexion angle of the ankle joint during the gait cycle.

The ranges of motion in the experimental group were smaller compared to those of the CG by 4.5° (KF1) and 2.9° (KF2), respectively. This manifested as a decrease in plantarflexion and an increase in dorsiflexion in both groups. Additionally, in the KF1 and KF2 groups, there was a limitation in joint angle changes during the loading response (LR) and a several-percent increase in plantarflexion relative to the CG. The largest differences are visible at 5% and 65% of the cycle, reaching a 5–7° limitation of movement in KF1 and KF2 in the direction of plantarflexion. This trend means that after the intervention, the subjects achieved a slightly greater dorsiflexion of the foot during the stance phase than healthy individuals while still having a limited range of plantarflexion in the push-off phase. Despite the increase in dorsiflexion, the general shape of the curve remained close to the reference value, and the key difference in KF2 was the persistent smaller range of plantarflexion at the end of the stance phase (Figure 3a). In the analysis of the difference modulus values, it should be noted that a reduction in the difference modulus of the KF2 profile was observed at approx. 55–60% of the cycle, with a decrease in the difference modulus value from 7.5% to 4%. Another area of improvement in results compared to KF1 was the mid-swing phase at 75–90% of the cycle, with a decrease in the difference modulus value by 2%. A slight deterioration of results occurred at approx. 10% of the cycle, with an increase in the difference modulus value by approx. 1%. Both the KF1 and KF2 curves differed the most at approx. 55% of the cycle, at a level of approx. 5.5%.

Below is a summary of the changes along with the interpretation of the result. In the “effect size” column, absolute values are provided in degrees or percentage points of the difference modulus because statistical significance tests were not used in the study (Table 4).

## 4. Discussion

The descriptive and exploratory nature of the study means that the observed changes in the profiles should be interpreted as temporal relationships co-occurring with the intervention, rather than as evidence of its effectiveness.

The results of the present study indicate that a four-week gait re-education program using the PRODROBOT gait trainer resulted in selective changes in lower-limb kinematics in the sagittal plane in children with cerebral palsy. These changes did not represent a global normalization of the gait pattern but rather concerned specific joints and phases of the gait cycle. The most pronounced direction of improvement was observed in the ankle joint and, to a lesser extent, the knee joint, whereas the hip joint movement pattern after the intervention remained similar to baseline.

This pattern of results is consistent with the current body of knowledge regarding robot-assisted gait training in children with cerebral palsy. Contemporary systematic reviews indicate that RAGT may improve gait function, balance, and selected spatiotemporal parameters; however, its effect on detailed kinematic parameters remains inconclusive [15,27,28,29]. This means that simply increasing the number of gait cycle repetitions does not necessarily lead to the restoration of a physiological movement pattern. In children with cerebral palsy, the gait pattern is the result of the coexistence of primary neurological impairments, secondary musculoskeletal changes, and established movement compensations [6,7].

The smallest changes were observed in the hip joint. The F1 index for the KF2 vs. KF1 comparison was 2.0%, whereas F2 reached 95.6%, indicating a very high similarity between the profiles before and after the intervention. At the same time, comparison with the control group demonstrated the persistence of a substantial deviation from the physiological pattern. Hip flexion–extension curves in both measurements remained shifted toward increased flexion, and physiological hyperextension was not observed during the terminal stance phase. In practice, this means that the therapy did not substantially change the movement strategy of the hip joint.

From a biomechanical perspective, the lack of hip extension during the terminal stance phase is important because it limits step length, disrupts the smooth forward transfer of the body’s center of mass, and may increase compensatory loading of the knee and ankle joints. In children with cerebral palsy, a persistent flexed hip posture frequently coexists with increased anterior pelvic tilt, weakness of the hip extensors, shortening of the hip flexor muscles, and impaired trunk control [30,31]. For this reason, improvement in hip kinematics probably requires not only repetitive gait training but also simultaneous work on pelvic control, trunk stabilization, hip extensor strength, and hip extension range of motion.

Therefore, the results concerning the hip joint should be interpreted with caution. The absence of a clear improvement does not indicate that the applied therapy was ineffective but rather suggests that the proximal segment of the lower limb is less susceptible to short-term modification than the distal segments. Similar difficulties have been described in studies using the Lokomat system, in which functional improvement did not always translate into significant changes in gait kinematic parameters [28].

A different pattern was observed in the knee joint. The KF2 vs. KF1 comparison met the criteria for profile similarity, indicating that the overall shape of the curve after therapy remained similar to baseline. At the same time, the difference module analysis demonstrated a local reduction in deviations relative to the control group, particularly during the initial stance phase, around 55–60% of the gait cycle, and during the terminal swing phase. This indicates that, despite the absence of a fundamental reconstruction of the movement pattern, partial improvement in knee control occurred during phases that are particularly important for stability and preparation of the limb for ground contact.

The persistence of increased knee flexion compared with the control group throughout a substantial portion of the gait cycle may be interpreted as a component of crouch gait. Crouch gait is one of the more common gait patterns in children with spastic forms of cerebral palsy and is characterized by increased hip and knee flexion together with excessive ankle dorsiflexion during the stance phase [23]. This pattern substantially increases the demand for the knee extensor moment, overloads the quadriceps femoris muscle, and may lead to an increased energy cost of walking [32]. Therefore, even a moderate improvement in knee alignment during the stance phase may be clinically meaningful, particularly if it reduces the degree of stance-phase knee flexion. However, it should be emphasized that the present study did not demonstrate complete normalization of knee function. Differences relative to the control group were still present. This suggests that therapy using the PRODROBOT system may have improved selected aspects of movement coordination but did not eliminate the biomechanical causes responsible for the persistent flexed limb posture. These causes may include deficits in knee extensor strength, impaired muscle activation, shortening of the hamstring muscles, or insufficient plantar flexor function during the terminal stance phase.

The most interesting results were obtained for the ankle joint. After the intervention, the ankle joint range of motion approached the reference values, and the difference relative to the control group decreased from 4.5° to 2.9°. Furthermore, the difference module analysis demonstrated improvement around 55–60% of the gait cycle and during the mid-swing phase. This indicates that after the intervention, a directional change toward the reference pattern was observed in the control of foot positioning during both the terminal stance phase and the swing phase. The importance of this finding is considerable because the ankle joint plays a fundamental role in generating propulsion during gait. The plantar flexor muscles are responsible for body support, forward progression of the center of mass, and initiation of the swing phase [8,9]. Limitation of plantar flexion during the terminal stance phase reduces push-off efficiency and may force compensatory activity of the proximal segments. In the study group, despite improvement in the ankle joint profile, limitation of plantar flexion during push-off was still present. This indicates that the improvement concerned foot positioning and movement control rather than complete restoration of the propulsion mechanism. It is worth noting that the improvement in the ankle joint may have resulted from the specific characteristics of robotic training. The repetitive, rhythmic stepping sequence promotes the consolidation of appropriate foot positioning during the stance and swing phases. However, it may not be sufficient to restore active plantar flexor strength, particularly when the device partially guides limb movement. Therefore, in clinical practice, robotic gait training should be combined with calf muscle strengthening exercises, eccentric–concentric training, and reactive weight-bearing exercises [33].

The results of the present study are consistent with current systematic reviews. Cortés-Pérez et al. demonstrated that RAGT may be more effective than conventional therapy in improving walking speed, walking distance, and selected motor functions, although not all gait parameters show significant improvement [27]. Wang et al., in a network meta-analysis, indicated that RAGT has beneficial effects on gait function and balance in patients with cerebral palsy, whereas its effectiveness regarding walking speed and spasticity remains uncertain [15]. Lim et al. further emphasized that the effects depend on the type of device and the characteristics of the applied training [29]. Of particular importance is the comparison of the present findings with the study by Choi et al., in which a portable overground exoskeleton was used in 90 children with cerebral palsy. The authors demonstrated significant improvements in motor function, balance control, and the Gait Deviation Index following robotic training [34]. The differences between those findings and the results of the present study may be explained by differences in device design, intervention duration, and the application of the assist-as-needed strategy. Overground systems may engage active postural control to a greater extent, whereas treadmill-based and more movement-guiding devices provide high repeatability but may limit the independent generation of propulsive moments. In healthy participants, Chambers et al. found that a variable-stiffness treadmill increased activity in muscles around the hip and knee while reducing heel-strike impact forces [35]. This provides a mechanistic example of how robotic device design can alter muscular demands, but it does not establish the effectiveness of such training in children with cerebral palsy.

Against the background of the existing literature, the results of the present study should be regarded as moderately favorable. Complete normalization of gait was not demonstrated; however, directional improvement was observed during biomechanically important phases of the gait cycle. This is consistent with the assumption that, in children with cerebral palsy, the effectiveness of therapy should not be assessed solely by the achievement of a normal gait pattern. A more realistic goal is the reduction of the most burdensome gait deviations, improvement of stance stability, increased safety during the swing phase, and reduction of proximal compensations. An important strength of the present study is the use of three-dimensional gait analysis and evaluation of complete angular waveforms. Current clinical guidelines emphasize the importance of instrumented gait analysis in children with cerebral palsy because it enables identification of gait deviations, treatment planning, and evaluation of intervention effectiveness [36]. In the present study, the analysis was not limited to spatiotemporal parameters but also included angular waveforms throughout the entire gait cycle, making it possible to identify local changes during specific phases. This is particularly important because average walking speed or step length may fail to reveal subtle but clinically relevant changes in joint mechanics.

The use of the F1 and F2 indices made it possible to provide a synthetic assessment of waveform similarity; however, interpretation of these indices requires caution. A high F2 value does not always indicate clinical normalization of the movement pattern because the waveforms may be highly similar before and after therapy while still remaining substantially different from the reference pattern. This situation was observed particularly in the hip joint. Therefore, similarity indices should be interpreted together with the analysis of difference modules and phase-by-phase assessment of the gait cycle. From a practical perspective, the results indicate that PRODROBOT may be a valuable tool supporting gait re-education, particularly in consolidating a repetitive stepping pattern and improving control of the distal segments of the lower limb. However, it should not replace comprehensive physiotherapy. In children with cerebral palsy, robotic training should be combined with interventions targeting muscle strength, selective motor control, range of motion, pelvic and trunk stabilization, and functional overground training. Outcome-specific responses have also been reported outside cerebral palsy: Chatzopoulos et al. observed improved joint position sense after six weeks of fascia-oriented training in adult dancers, without significant between-group differences in force sense or postural control [37]. This indirect comparison illustrates why changes in joint-level measures should be evaluated separately from balance and functional outcomes; it does not support extrapolating that intervention to children with cerebral palsy.

### 4.1. Study Limitations

A limitation of the study is the small size of the experimental group, resulting, among other factors, from the withdrawal of some participants who were unable to complete the therapy, as well as from the organizational consequences of the pandemic. This limits the generalizability of the obtained results. A second limitation is the quasi-experimental pre–post design, without randomization and without a group of children with cerebral palsy receiving conventional therapy only. Therefore, it cannot be unequivocally determined what proportion of the observed changes resulted from the effect of the device and what proportion resulted from the natural variability of gait, continued rehabilitation, or a learning effect during the repeated assessment. The kinematic assessment was not blinded. The same team conducted the intervention, data recording, and data processing, which, in the absence of randomization, constitutes a potential source of systematic bias. The use of automatic gait cycle event detection and quantitative *f*_1_ and *f*_2_ factors relatively reduces this risk. The analysis was limited to sagittal plane kinematics: kinetic parameters, i.e., ground reaction forces and joint moments and powers, as well as surface electromyography, were not recorded. This makes it impossible to determine whether the observed changes in angular profiles result from an alteration in the muscle activation pattern and the generated driving torque or merely from a change in the position of the limb segments guided by the device.

The involvement of natural developmental changes in the differences observed between KF1 and KF2 cannot be completely ruled out. In children at GMFCS level III, gross motor function reaches a plateau after 7–8 years of age and then shows a downward trend [24], while gait parameters gradually deteriorate in the following years. Resolving this issue requires a randomized study with a parallel group of children with CP receiving exclusively conventional therapy.

### 4.2. Future Directions

Future studies should include larger groups of children with cerebral palsy, preferably stratified according to gait pattern type and degree of spasticity. Stratification should include the sagittal plane gait pattern subtypes according to Rodda and Graham [23] for bilateral forms and according to Winters et al. [38] for hemiplegic forms because the response to gait re-education using robotic devices may differ between patterns. It would be advisable to use a randomized study design comparing the PRODROBOT system with conventional therapy, treadmill training, and other robotic systems. Studies with longer follow-up periods would be particularly valuable, allowing assessment of the durability of the effects after 3, 6, and 12 months. The analysis should also be expanded to include kinetics, surface electromyography, the energetic cost of gait, and global indices such as the Gait Deviation Index (GDI) or the Gait Profile Score (GPS).

## 5. Conclusions

1. The four-week gait re-education program using the PRODROBOT gait trainer resulted in selective changes in lower-limb kinematics in the sagittal plane in children with cerebral palsy; however, due to the single-arm study design, these changes cannot be unequivocally attributed to the intervention itself. The most pronounced changes were observed in the ankle joint and in selected phases of knee joint movement, whereas the hip joint movement pattern remained essentially unchanged.
2. The use of complete angular waveforms together with profile similarity and difference indices (F1 and F2) made it possible to detect local changes in individual phases of the gait cycle that would not have been identified based solely on spatiotemporal parameters. The findings confirm the usefulness of three-dimensional gait analysis in evaluating the effects of therapy in children with cerebral palsy.
3. The obtained results indicate that training using the PRODROBOT device may constitute a valuable component of comprehensive gait rehabilitation; however, it does not by itself lead to complete normalization of the locomotor pattern. Improvement in selected kinematic parameters does not eliminate the persistent biomechanical impairments characteristic of children with cerebral palsy.
4. In light of the obtained findings, robotic gait re-education should be considered as a complement to a comprehensive rehabilitation program including interventions aimed at improving muscle strength, range of motion, selective motor control, and trunk and pelvic stabilization.
5. Confirmation of the clinical effectiveness of therapy using the PRODROBOT device requires further studies involving larger groups of participants, with randomization, an appropriate control group, and longer follow-up periods to assess the durability of the achieved effects.

## Figures and Tables

**Table 1:** Comparison of spatiotemporal parameters.

STP (Spatiotemporal Parameters)	KF1		KF2		CG	
	$\bar{x}$	s	$\bar{x}$	s	$\bar{x}$	s
WS (walking speed) [ms^−1^]	0.91	0.07	0.93	0.07	1.01	0.08
STRT (stride time) [s]	1.04	0.06	1.02	0.07	1.07	0.05
STRL (stride length) [m]	0.95	0.08	0.91	0.07	0.94	0.09
CAD (cadence) [Hz]	1.92	0.12	1.96	0.13	1.85	0.08
DSAP (double support time) [%]	21	2.4	22	2.5	25	2.2
SSAP (single support time) [%]	39	2.1	38	4.2	37	3.3
OFO (foot off) [%]	61	1.7	61	1.1	62	1.2

**Table 2:** Comparison of anthropometric values with homogeneity tests.

Parameter	Intervention	Control	t	df	*p*
Age [years]	9.60 ± 2.45	10.10 ± 2.42	−0.52	18.5	0.609
Body height [m]	1.31 ± 0.13	1.34 ± 0.12	−0.60	17.5	0.555
Body mass [kg]	31.25 ± 9.47	32.36 ± 7.27	−0.32	15.0	0.752
Pelvic width [m]	0.236 ± 0.030	0.241 ± 0.028	−0.43	17.6	0.671
BMI [kg/m^2^]	17.63 ± 2.00	17.85 ± 2.10	−0.27	19.5	0.787

BMI—body mass index; t—Welch’s *t*-test; df—degrees of freedom; *p*—probability value; Two-tailed Welch *t*-tests showed no significant between-group differences (*p* > 0.55; d < 0.25).

**Table 3:** Values of the similarity and difference indices of the analyzed pairs of variables.

Variable Pairs—Sagittal Plane	F1	F2
ANKLE DOR/PLA_KF2/ANKLE DOR/PLA_KF1	22.6	80.3
ANKLE DOR/PLA_KF1/ANKLE DOR/PLA_CG	44.7	70.4
ANKLE DOR/PLA_KF2/ANKLE DOR/PLA_CG	50.4	70.3
KNEE FLEX/EXT_KF2/KNEE FLEX/EXT_KF1	5.7	80.5
KNEE FLEX/EXT_KF1/KNEE FLEX/EXT_CG	22.2	61.3
KNEE FLEX/EXT_KF2/KNEE FLEX/EXT_CG	17.4	61.4
HIP FLEX/EXT_KF2/HIP FLEX/EXT_KF1	2.0	95.6
HIP FLEX/EXT_KF1/HIP FLEX/EXT_CG	54.1	50.2
HIP FLEX/EXT_KF2/HIP FLEX/EXT_CG	53.0	50.2
ANKLE DOR/PLA_KF2/ANKLE DOR/PLA_KF1	22.6	80.3
ANKLE DOR/PLA_KF1/ANKLE DOR/PLA_CG	44.7	70.4
ANKLE DOR/PLA_KF2/ANKLE DOR/PLA_CG	50.4	70.3
KNEE FLEX/EXT_KF2/KNEE FLEX/EXT_KF1	5.7	80.5
KNEE FLEX/EXT_KF1/KNEE FLEX/EXT_CG	22.2	61.3
KNEE FLEX/EXT_KF2/KNEE FLEX/EXT_CG	17.4	61.4
HIP FLEX/EXT_KF2/HIP FLEX/EXT_KF1	2.0	95.6
HIP FLEX/EXT_KF1/HIP FLEX/EXT_CG	54.1	50.2
HIP FLEX/EXT_KF2/HIP FLEX/EXT_CG	53.0	50.2

KF1—pre-intervention; KF2—post-intervention; CG—control group. Profiles were considered similar when the following were met simultaneously: F1 < 15% and 50 < F2 ≤ 100.

**Table 4:** Summary of kinematic changes.

Joint	Gait Cycle Phase (% of Cycle)	Direction of Change (KF1 → KF2)	Magnitude of Effect	Interpretation
Hip	Entire cycle (0–100%)	No change	*f*_1_ = 2.0%; *f*_2_ = 95.6 (KF2 vs. KF1)—profiles meet the similarity criteria	The movement strategy of the hip joint was not modified
Hip	Entire cycle—relative to CG	No change	*f*_1_ 54.1% → 53.0% (Δ = −1.1 pp); *f*_2_ 50.2 → 50.2 (Δ = 0.0)	Persistent deviation from the physiological pattern
Hip	Loading response, LR (0–10%)	Improvement	Reduction of the mean difference relative to CG by 1–2°	Change below the threshold of clinical relevance
Hip	Terminal stance, TST (30–50%)	No change	Persistent flexion approximately 10° greater than CG; absence of hyperextension	Limitation of step length and of center-of-mass progression
Knee	Entire cycle—relative to CG	Improvement	*f*_1_ 22.2% → 17.4% (Δ = −4.8 pp); *f*_2_ 61.3 → 61.4 (Δ = +0.1)	Reduction of the relative error in curve shape
Knee	Entire cycle (KF2 vs. KF1)	No change in curve shape	*f*_1_ = 5.7%; *f*_2_ = 80.5—profiles meet the similarity criteria	Local changes without a change in the overall pattern
Knee	Loading response, LR (0–5%)	Improvement	Reduction of the difference modulus relative to CG by 2–3 pp (from a maximum of 12%)	Improved knee control during weight acceptance
Knee	Terminal stance, TST (55–60%)	Improvement	Reduction of the difference modulus relative to CG by 2–3 pp	Improved stability during single-limb support
Knee	Terminal swing, TSW (95–100%)	Improvement	Reduction of the difference modulus relative to CG by 2–3 pp	Better preparation of the limb for ground contact
Knee	Stance phase (0–60%)	No change	Persistent increased flexion; range of motion 46° → 51°	Persistent crouch gait component
Knee	Mid-swing, MSW (75–90%)	No change	Smallest differences relative to CG across the entire cycle	Phase closest to the norm at baseline
Ankle	Entire cycle—range of motion	Improvement	Range-of-motion deficit relative to CG 4.5° → 2.9° (Δ = +1.6°)	Change within the limits of measurement uncertainty
Ankle	Entire cycle (KF2 vs. KF1)	Change in curve shape	*f*_1_ = 22.6%; *f*_2_ = 80.3—the *f*_1_ criterion is not met	The greatest profile reorganization among the three joints
Ankle	Terminal stance/pre-swing (55–60%)	Improvement	Difference modulus 7.5% → 4.0% (Δ = −3.5 pp)	The strongest local effect in the entire study
Ankle	Mid-swing, MSW (75–90%)	Improvement	Reduction of the difference modulus by 2.0 pp	Improved control of foot positioning during swing
Ankle	Loading response, LR (approx. 10%)	Deterioration	Increase of the difference modulus by approximately 1.0 pp	Change within the limits of measurement uncertainty
Ankle	Entire cycle—relative to CG (f_1_)	Deterioration	*f*_1_ 44.7% → 50.4% (Δ = +5.7 pp); *f*_2_ 70.4 → 70.3	Increased dorsiflexion during stance despite the improvement in range of motion

KF1—pre-intervention measurement; KF2—post-intervention measurement; CG—control group; LR—loading response; TST—terminal stance; TSW—terminal swing; MSW—mid-swing; pp—percentage points.

## Data Availability

The original contributions presented in the study are included in the article. Further inquiries can be directed to the corresponding authors.
